# Letter-By-Letter Reading: Natural Recovery and Response to Treatment

**DOI:** 10.1155/2005/413962

**Published:** 2006-02-27

**Authors:** Pélagie M. Beeson, Joël G. Magloire, Randall R. Robey

**Affiliations:** ^1^Department of SpeechLanguageand Hearing Sciences and Department of Neurology The University of ArizonaTucsonAZUSA; ^2^New York Eye and Ear InfirmaryNew YorkNYUSA; ^3^Communication Disorders Program University of VirginiaCharlottesvilleVAUSA

**Keywords:** Alexia, dyslexia, letter-by-letter reading, treatment

## Abstract

The present investigation provides a longitudinal study of an individual (RB) with acquired alexia following left posterior cerebral artery stroke. At initial testing, RB exhibited acquired alexia characterized by letter-by-letter (LBL) reading, mild anomic aphasia, and acquired agraphia. Repeated measures of reading accuracy and rate were collected for single words and text over the course of one year, along with probes of naming and spelling abilities. Improvements associated with natural recovery (i.e., without treatment) were documented up to the fourth month post onset, when text reading appeared to be relatively stable. Multiple oral reading (MOR) treatment was initiated at 22 weeks post-stroke, and additional improvements in reading rate and accuracy for text were documented that were greater than those expected on the basis of spontaneous recovery alone. Over the course of one year, reading reaction times for single words improved, and the word-length effect that is the hallmark of LBL reading diminished. RB's response to treatment supports the therapeutic value of MOR treatment to in LBL readers. His residual impairment of reading and spelling one-year post stroke raised the question as to whether further progress was impeded by degraded orthographic knowledge.

## 1. Recovery from letter-by-letter reading

Letter-by-letter (LBL) reading has been called the hallmark of pure alexia [8,39]. It refers to the reading strategy whereby sequential identification of component letters (often aloud) is necessary to facilitate word recognition. Consequently, the time to name a word in LBL reading is a function of word length. Increases in latency of 500 milliseconds to several seconds or more have been reported for each additional letter [2,5, 15,37,42]. This increase is far more exaggerated than the word length effect reported in some neurologically intact individuals which is on the order of 6 to 63 milliseconds [24].

While consensus is lacking regarding the precise nature of the impairment that results in LBL reading, it is generally considered a peripheral (as opposed to central) reading impairment [9]. The reading difficulty has been characterized as the result of damage to early visual analysis or letter identification processes [1,3, 14,17]. Alternatively, it has been thought to reflect a disconnection of the visual input from the orthographic input lexicon [22,37]. Regardless of the underlying cause, all individuals labeled as LBL readers show a word length effect in reading. They may differ with regard to accuracy of letter identification, which influences the ability to use the letter-by-letter reading strategy effectively [34,37,42]. In addition, LBL readers may diverge regarding the integrity of more central cognitive processes necessary for processing written language. Some LBL readers use a phonological strategy to decode the input from their LBL reading, resulting in a surface alexia profile [45]. Phonological and

ISSN 0953-4180/05/$17.00 © 2005 – IOS Press and the authors. All rights reserved deep alexia also have been documented in some LBL readers indicating additional damage to phonological procedures [11,18,31]. By definition, pure alexia has been characterized as an acquired reading impairment in the absence of significant writing impairment; however, LBL may also be accompanied by spelling impairment [17,22,37]. In such cases, LBL is not truly a pure alexia, but is more accurately described as a form of alexia *with* agraphia. It is plausible that differences in the patterns of impairment in individual LBL readers will influence the course of their recovery and responsiveness to particular treatment programs.

Most studies of LBL reading have focused on the nature of the deficit, while few studies of LBL reading have examined the evolution of the reading deficit over time. A better understanding of recovery processes is pertinent to establishing efficient and cost-effective programs for rehabilitation of acquired alexia. A review of the literature yielded 14 studies of LBL reading that provided longitudinal data for individual subjects (see Table 1). Eleven of those studies reported on recovery in response to behavioral treatment, and included a total of 12 LBL readers. Three longitudinal studies did not involve treatment but provided information about natural (i.e., spontaneous) or unaided recovery.

### *1.1. Natural recovery of letter-by-letter reading*

Although few in number, studies of natural recovery of abilities in LBL readers (i.e., without treatment) show several patterns of change over time. Table 1 provides a summary of changes on selected behavioral measures from three longitudinal studies. A review of these studies reveals that without treatment LBL readers may show improvement (but not necessarily recovery) in their letter naming ability [23,34], single-word reading accuracy [23,30,34], reading reaction time [8], and text reading accuracy and rate [23]. When a letter naming deficit was present, improvement in single-word reading accuracy occurred in parallel with improved letter identification [34]. Newcombe's patient MB showed full recovery of letter naming between 20 and 30 weeks post onset [34], and Case #5 reported by Hecaen et al. [23] completely recovered letter naming by about 6 weeks. MB's recovery of letter naming was followed by improvement in single-word reading accuracy on a word list consisting of short (i.e., 3 letter) words. Hecaen et al. [23] also reported that improved letter naming was followed by improved single-word reading accuracy. A patient reported by Marshall et al. [30] showed improvements in single-word reading accuracy that were paralleled by improved object naming performance. With regard to text reading, Hecaen et al. [23] reported that their Case #5 read text slowly, but accurately, at 47 weeks, and was reading text at nearly normal rates by 69 weeks (although no actual reading rates were provided). Across these studies, a word length effect on accuracy or reaction time appeared to persist in all subjects, suggesting that the qualitative character of LBL reading remained consistent over time.

### *1.2. Recovery in response to treatment*

Various studies have documented changes in reading ability by LBL readers over time in the context of reading treatments. Table 1 summarizes the changes in selected behavioral measures by LBL readers as reported in the available treatment studies. Several studies focused on treatment for single-word reading ability [2,19,20,28,29,32]. Among these studies, improvements were documented for single-word reading accuracy [19,28], single-word reaction times for practiced word lists [19,45], single-word reaction times for novel (unpracticed) word lists [2,20,28], and sentence or text reading rate [20,28,29]. Of those studies that mentioned the status of word length effects at the end of treatment, all found persistent word length effects on reading reaction time. The persistence of the qualitative character of LBL reading was documented for a period up to 5 years post onset [45].

Several studies described the effectiveness of a treatment program using Multiple Oral Reading (MOR), a procedure that focuses on repeated re-reading of text in order to improve text reading rate [5,6,32,33,41]. Moyer [33] first reported the use of the MOR technique in a case study in which she successfully improved the reading speed of a man who showed the characteristics of LBL reading. Prior to, and concurrent with, the MOR treatment program, the participant received treatment to improve letter discrimination and recognition. MOR homework involved re-reading a given text 30 minutes a day for one week. Weekly reading rate was measured and a new passage was assigned for MOR during the following week. Improved reading rate for new text was documented over two months. While Moyer [30] reported overt LBL reading by her subject at initial evaluation, she did not report on the presence or absence of a word length effect following treatment. Improved text reading rate with MOR treatment has been documented for other pure alexic patients with LBL reading [5,31,41], as well as alexic

Table 1
Summary of performance on selected behavioral measures for alexic readers who exhibited word length effects on reading

| | Letter naming | Single-word reading accuracy | Word length effect – accuracy | Single-word reaction time | length effect – reaction time | Text reading rate |
|---|---|---|---|---|---|---|
| *Unaided recovery* | | | | | | |
| Marshall et al. [30][1] MB | ↑ | ↑ | →↑ | | | |
| Hecaen et al. [23] Case #5 | ↑ | ↑ | → | | | ↑ |
| Behrmann et al. [8] DS | OK | OK | | ↑ | →↑ | |
| *Aided Recovery* | | | | | | |
| Lott & Friedman [28] DL | ↑ | ↑ | →↑ | ↑ | →↑ | ↑ |
| Moyer [33]* | ↑ | → | | ↑ | | ↑ |
| Tuomainen & Laine [41]* HT | | ↑ | ↑ | ↑ | →↑ | ↑ |
| Tuomainen & Laine [41]* TT | | | | | → | ↑ |
| Beeson [5]* HL | OK | → | → | → | → | ↑ |
| Wilson [45] AW | OK | → | | ↑ (trained)<br>→ (untrained) | →↑ | ↑ |
| Arguin & Bub [2] DM | ↑ (RT) | → | → | ↑ | →↑ | |
| Gonzalez-Rothi & Moss [20] | | ↑ | | ↑ | | ↑ |
| Moody [31] LM & SK | OK | → | | → | → | ↑ |
| Maher et al. [29] VT | → | OK | | → | → | ↑ (sentences) |
| Beeson & Insalaco [6]* SV & TD | OK | OK | | ↑ | ↑ | ↑ |
| Friedman & Lott [19] RS | OK | ↑ (trained) | | →↑ | →↑ | |

Note: OK = unimpaired; ↑= Improved; →= Persistent effect; →↑= Persistent effect, but showed improvement.
*Indicates MOR treatment.
[1]Also discussed in [34,35].

patients with additional reading and language impairments [6,31]. Despite improved reading rates, word length effects typically persisted for the LBL readers in these studies, with the exception of two alexic subjects in whom the word length effect resolved by the end of treatment [6].

In summary, treatment studies have shown that text reading improved in response to both single-word and text reading treatments. MOR treatment was accompanied by treatment of letter naming and single-word recognition in the studies by Moyer [33] and Tuomainen and Laine [41], so that the effectiveness of MOR treatment alone was not entirely clear. A word length effect, albeit reduced in some cases, persisted in the face of improvements in single-word and text reading in both aided and unaided recovery in most participants. Thus, regardless of improvements in other aspects of reading ability, it appears that the characteristic feature of LBL reading (i.e., word length effects on single-word reading) typically persists, suggesting that access to orthographic representations remains impaired to some extent.

To date, the literature does not provide a clear picture of what aspects of acquired alexia are likely to improve without treatment, and what impairments respond to MOR treatment. We report here on a case that offered the opportunity to document recovery of acquired reading impairment in a LBL reader over its natural course (i.e., without treatment), as well as in response to MOR treatment. Reading performance was examined weekly with measurements of reading rate and accuracy for text. In addition, periodic measurements of letter naming ability and single-word reading accuracy and reaction time were performed to provide insight into the nature of underlying deficits.

## 2. Case report

RB was a 59-year-old, right-handed man with a history of hypertension, diabetes and degenerative joint disease who suffered a left hemisphere stroke. A hemorrhagic infarct was confirmed by MRI head scan in the distribution of the left posterior cerebral artery involving inferior temporal and occipital lobes, and sparing the medial occipital lobe and calcarine cortex (see Fig. 1). The stroke resulted in impaired reading and writing ability, mild anomia, and an upper right quadrantanopia. RB was a high school graduate who had been employed as a salesperson. He was forced to retire subsequent to the stroke. He reported being an avid reader before his stroke, and he occasionally enjoyed

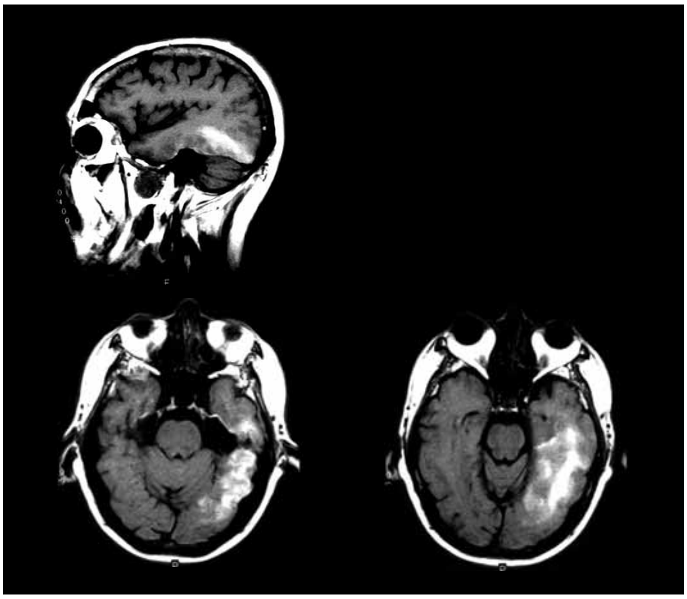

Fig. 1. Acute T-1 weighted MRI scan of RB showing hemorrhagic infarct in the left inferior temporo-occipital region.

writing poetry. He expressed great frustration with his acquired reading difficulty, as pleasure reading was one of his hobbies.

### 2.1. Initial evaluation and natural recovery

An initial language assessment was carried out over two sessions at 5 and 7 weeks post-stroke. RB achieved a score of 93.2 on the *Western Aphasia Battery* (WAB) [27]. His language profile was consistent with mild anomic aphasia characterized by good auditory comprehension and repetition, with mild word finding difficulty. Follow-up testing with the *Boston Naming Test* (BNT) [25] at 10 weeks post-stroke confirmed the naming impairment (43/60 items correct).

RB's reading and spelling abilities were tested at the initial evaluation as detailed below, and he was evaluated on a weekly basis to monitor his reading performance with the intention of documenting the course of natural recovery (i.e., without treatment). Treatment was to be initiated when gains in text reading rate were no longer observed with natural recovery. The pre-treatment period extended for 12 weeks (from 9 to 20 weeks post-stroke).

### 2.2. Letter naming

At five weeks after his stroke, RB showed some difficulty naming letters. He correctly named 19 of 26 lowercase and 16 of 26 uppercase letters. By seven weeks post-stroke, letter naming improved with RB naming 24/26 uppercase and 22/26 lowercase letters correctly (see Table 2). Letter naming was reassessed at 10 weeks post-stroke, and was approximating normal performance with RB correctly naming 25/26 uppercase letters and 26/26 lowercase letters, for a total of 51/52 letters correct (see Table 2). When tested at 13 weeks post-stroke, letter naming remained nearly normal with 25/26 uppercase and 24/26 lowercase letters named correctly. By 15 weeks post-stroke, letter naming was at 100% for both upper- and lower-case letters.

### 2.3. Single-word reading

Single-word reading ability was assessed initially and throughout the ensuing year using selected subtests from the *Psycholinguistic Assessment of Language Processing in Aphasia* (PALPA) [26]. Single words were presented in 24-point font on a 38-cm computer screen using the Superlab program [12]. Reaction times and accuracy were recorded for each word so that effects of word length, imageability, frequency, and grammatical class could be examined. Response times (in milliseconds) were measured from the time of visual presentation of words to the initiation of RB's oral response as signaled by experimenter button press. Reaction time analyses were computed for the correct reading responses only.

Table 2
RB's accuracy (in percent correct) on tests of letter naming, single-word reading and spelling (arranged vertically by time of administration)

| Weeks post-stroke | Letter name | Read aloud | | Written spelling | | | | Oral naming |
|---|---|---|---|---|---|---|---|---|
| | P-22 | P-31 | P-32 | P-31 | P-32 | P-53 | P-36 | BNT |
| 5 | 67% | 59% | | | | 70% | | |
| 7 | 88% | | 75% | 53% | | | | |
| 10 | 98% | | | | | | | 72% |
| 11 | | | 94% | | | | | |
| 12 | | 91% | | | | | | |
| 13 | 94% | | | | | 80% | | |
| 14 | | | | | 66% | | 88% | |
| 15 | 100% | | | 63% | | | | 75% |
| 23 | | | | 84% | | | | |
| 26 | | 95% | | | | | | |
| 29 | | | | | | | | 78% |
| 30 | | | 96% | | | | | |
| 40 | | 95% | 96% | 71% | 78% | 93% | 75% | 78% |
| 52 | 96% | | | 66% | 75% | | | 78% |
| 54 | | 98% | 99% | | | 93% | 89% | |

P-22 = PALPA 22, Letter naming.
P-31 = PALPA 31, List controlled for Imageability-Frequency ($n$ = 80).
P-32 = PALPA 32, List controlled for Grammatical Class ($n$ = 80).
P-53 = PALPA 53, Written picture naming ($n$ = 40).
P-36 = PALPA 36, Writing nonwords to dictation ($n$ = 24).
BNT = Boston Naming Test.

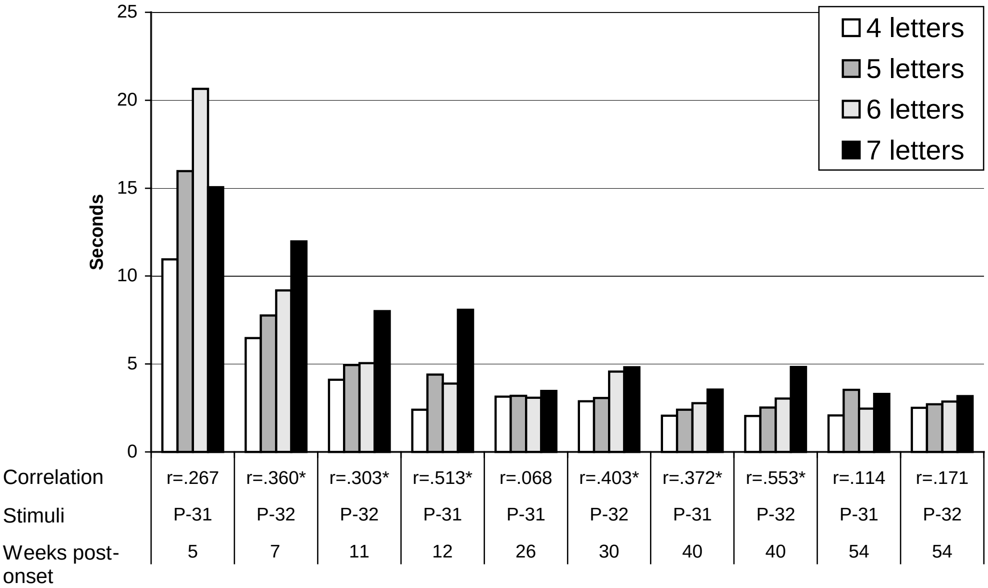

Fig. 2. RB's mean reading reaction times in seconds for words of 4, 5, 6, or 7 letters. Word lists from PALPA Subtest 31 (P-31) or PALPA Subtest 32 (P-32). Correlation = Pearson coefficient for word length by reaction time. *statistically significant at $p < 0.05$.

At initial testing, RB exhibited some overt letter-by-letter reading. At Week 5, his single word reading accuracy was impaired on the PALPA Subtest 31, controlled for imageability and frequency (47/80 correct; 59%). At Week 7, on the PALPA Subtest 32 controlled for grammatical class, RB read 60/80 correct (75%). On those lists, RB read high frequency words more accurately than low frequency words ($\chi^2 = 6.24$, $p < 0.02$), but no effect of imageability or grammatical class was observed. Reading errors on both word lists were characterized by visually similar words substituted for the target words (e.g., analogy → analog; concept →

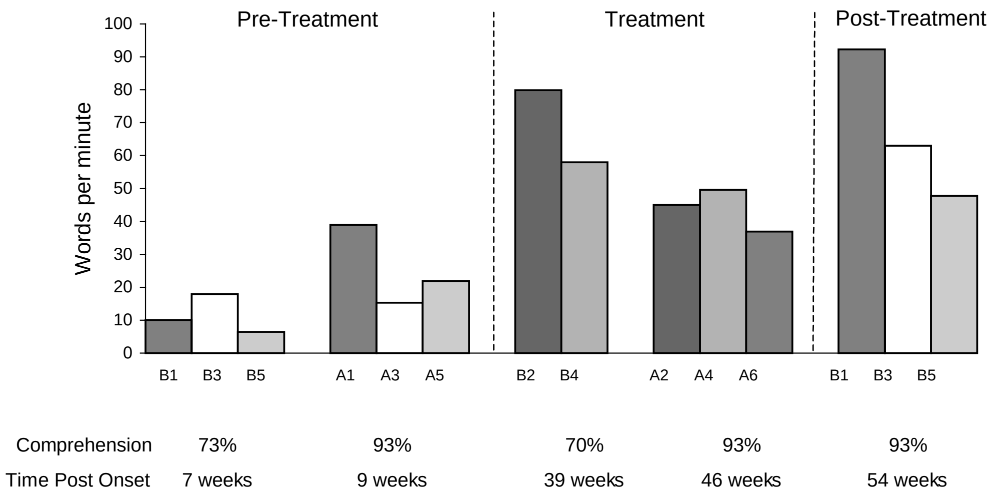

Fig. 3. RB's reading rates for text on the Gray Oral Reading Test-3. Passages from Form B or Form A, as indicated. Comprehension = response to multiple choice questions.

concert; satire → retire) for almost half of all reading errors (41%), with other error types including unrelated words (20%) and instances of no response (27%). RB's single-word reading reaction time was extremely slow, averaging 18 seconds (s.d. = 12.9 seconds) and 8 seconds (s.d. = 5.5 seconds) per word on Weeks 5 and 7, respectively. The effect of word length was not significant for reading accuracy, but the effect of word length on reaction time was significant at 7 weeks ($r$ = 0.360, $p$ = 0.012), with an average increase in reaction time of 1.7 seconds per letter (see Fig. 2).

Single-word reading was reassessed during two sessions at 11 and 12 weeks post-stroke (see Table 2). On the PALPA lists 32 and 31, RB's reading improved to 94% and 91% correct, indicating significant natural recovery of reading accuracy for single words , ($\chi^2$ = 10.67, $p$ = 0.001, $\chi^2$ = 22.53, $p <$ 0.001, respectively). The remaining errors were predominantly visually similar words (58%). Although overt LBL reading was rarely apparent at this time, a significant effect of word length on reading reaction time was present as shown in Fig. 2 at Weeks 11 and 12 ($r$ = 0.303, $p$ = 0.016; $r$ = 0.513, $p <$ 0.001, respectively).

### 2.4. *Text reading*

Oral reading of connected text was assessed nine weeks after RB's stroke using portions of the *Gray Oral Reading Test-3* (GORT-3), Form B [44], with other portions of the test reserved for future testing. His average reading rate on passages 1, 3, and 5 was 11.49 words per minute. This rate was markedly slower than neurologically intact adults who typically average 150 to 200 wpm when reading aloud [38]. Reading accuracy was also impaired with a mean of 9.8 uncorrected errors and 5.3 self-corrections per 100 words. Comprehension of this material was mildly impaired with RB correctly answering 73% of the 15 multiple choice questions correctly.

At nine weeks post stroke, text reading was assessed using the GORT-3, Form A. RB averaged 25.4 wpm per passage on the GORT-3 with a mean of 4.7/5 (93%) questions answered correctly per passage (see Fig. 3). His oral reading errors averaged 1.7 uncorrected errors and 2.3 self-corrections per 100 words. This performance reflected some improvements in reading rate and accuracy compared to 7 weeks post onset, and relatively normal reading comprehension.

RB's reading rate and accuracy were assessed during each weekly session beginning in the 9th week post-stroke using selected passages from *Scientific Research Associates Reading Laboratory* (SRA) [38]. SRA passages came from an estimated grade level of 2.4 and averaged 210 words in length. As shown in Fig. 4, RB's reading rate for the SRA text ranged from 14.8 to 25 words per minute and 1.7 to 5 errors per 100 words over the first three weeks. By week 14, RB's reading rate improved to approximately 40 words per minute on the SRA passages. During the period from 14 to 20 weeks post-stroke, reading rate for SRA passages fluctuated between 30 and 40 wpm, with an average accuracy of 3 uncorrected errors and 4 self-corrections per 100 words.

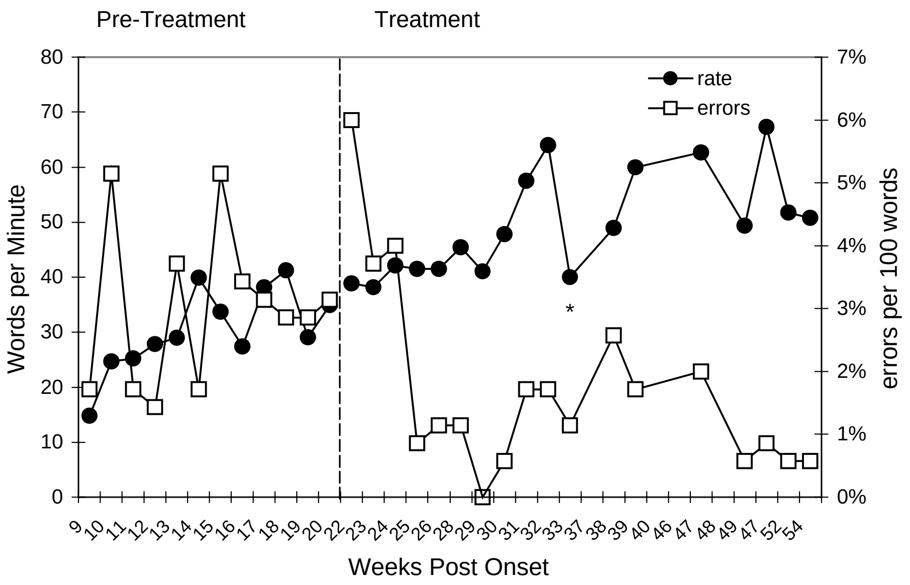

Fig. 4. RB's reading rate (words per minute) and uncorrected reading errors (per 100 words) when reading new text over the course of one year following stroke (during pre-treatment and treatment phases). * denates eye infection affecting visual acuity.

### 2.5. *Written spelling*

A sample of RB's writing before the stroke was obtained and confirmed relatively good premorbid spelling skills. Single-word written spelling was assessed using subtests from the PALPA (see Table 2). On a test of written picture naming (PALPA Subtest 53), RB spelled 16/20 (80%) regular words and 12/20 (60%) exception words correctly. Although not significant, these results were in the direction of a regularity effect on spelling. Spelling errors included phonologically implausible misspellings, such as *heare* for horse (42%) and phonologically plausible errors, such as *hart* for heart and *boll* for bowl (33%). At 7 weeks post-stroke, RB's written spelling of words was assessed with a word list controlling for imageability and frequency (PALPA Subtest 31). Spelling was impaired (42/80 correct; 53%) and revealed significant effects for frequency ($\chi^2 = 7.22$, $p = 0.007$) and imageability ($\chi^2 = 5.01$, $p = 0.025$). At that time, RB produced a preponderance of phonologically plausible errors (76% of all errors), reflecting reliance on nonlexical spelling strategies and consistent with a profile of lexical agraphia [4].

Single-word writing to dictation was reassessed at 13, 14, and 15 weeks post-stroke using three PALPA subtests: written picture naming (PALPA 53), a list controlling for imageability and frequency (PALPA 31), and a list controlling for grammatical class (PALPA 32). As shown in Table 2, RB's spelling accuracy showed some improvement from 53% at initial assessment to 63% ($\chi^2 = 1.637$, $p = 0.201$, ns). At that time, phonologically plausible errors were the most frequent type of spelling error, suggesting reliance on the nonlexical spelling route. While RB still exhibited an effect of imageability on his writing ($\chi^2 = 5.33$, $p = 0.021$), the frequency effect was no longer apparent. A list of nonwords (PALPA Subtest 36) was administered for writing to dictation and RB wrote 21/24 (88%) correctly, confirming that he had fairly well preserved ability to use sound-to-letter conversion strategies for spelling.

### 2.6. *Interpretation of pre-treatment recovery*

During this period of observation prior to treatment, RB showed fairly complete resolution of his mild letter naming impairment by 15 weeks post-stroke. Single-word reading accuracy also improved, but continued to be mildly impaired with reaction time affected by word length. Reading rate for text, which had been sampled most consistently, showed improvement up to about 14 weeks post-stroke, but did not show continued improvement from 14 to 20 weeks post-stroke. RB also had a relatively persistent impairment in single-word spelling that was best characterized as lexical agraphia. It was the reading impairment that was of primary concern to RB, and thus it was the focus of treatment.

### 2.7. Response to treatment

At 22 weeks post-stroke, reading treatment was initiated. RB was seen weekly over the course of 32 weeks, with some sessions missed due to minor illness, vacation, or conflicting appointments. During that time, the Multiple Oral Reading treatment (MOR) was implemented in order to improve his reading of text. The dependent variables of primary interest were reading rate and accuracy for new text. Periodic re-assessment of single-word reading, written spelling, and oral naming were conducted in order to provide additional information regarding his recovery.

### 2.8. Text reading

The MOR protocol was implemented in much the same manner as described by Beeson [5] and Beeson and Insalaco [6]. RB's reading rate and accuracy were initially determined for a given passage of text from the SRA reading series, and then that passage was assigned for practice at home. Homework consisted of repeated daily readings of the assigned SRA text for a minimum of 30 minutes. RB kept a log to maintain accountability for his home practice. He recorded the number of times that he read the homework passage and how much time he spent on MOR each day. The SRA materials were from levels 3.0 and 3.5 and averaged 410 words per passage (range = 350–470 words). During each therapy session, reading rate was measured for the practiced text as well as a novel (i.e., previously unread) passage. When the reading rate for a practiced text improved to the criterion level of 100 words per minute, a new passage was assigned as homework for the next week.

Over the first five weeks of MOR treatment RB averaged 41.7 wpm on novel texts with a range from 38.9 to 42.1 wpm. As shown in Fig. 4, this performance reflected a decrease in the variability of his reading rate compared to the 6 weeks immediately prior to initiation of MOR treatment and a drop in the frequency of errors, but did not reflect improvement in overall reading rate. It was hypothesized that RB might benefit from an increase in the target rate for practiced text from 100 to 120 wpm, so this change was implemented at 28 weeks post-stroke. Following the criterion change, RB's reading rate for novel text improved steadily from about 40 to 60 wpm over the next several weeks as shown in Fig. 4. By 32 weeks after his stroke, RB's novel text reading rate had reached an average of 64 wpm with a mean of 0.6 errors and 1.1 self-corrections per 100 words. It was notable that during this time, RB reported subjective improvement in his ability to engage in pleasure reading. He had a brief bout of peripheral eye problems that interfered with reading due to discomfort during Week 37. During the holiday season from weeks 41 to 45, RB continued his home program but did not attend weekly sessions at the clinic. Upon return from the holiday break, RB's reading rate fluctuated between 50 and 70 words per minute (see Fig. 4). Treatment was terminated at 54 weeks post-stroke as RB was changing his place of residence and could no longer attend therapy.

Visual inspection of Fig. 4 shows that over the course of treatment RB's reading rate increased as his error rate decreased. His mean reading rate during the pretreatment phase was 31.63 wpm (s.d. = 7.51) compared to 50.11 (s.d. = 8.98) over the course of treatment. The number of errors per 100 words decreased from pre-treatment (mean = 3.29; s.d. = 1.91) compared to the treatment phase (mean = 1.47; s.d. = 0.98). In order to more directly compare the relative change in performance during treatment versus spontaneous recovery, RB's change in level of performance during the first 3 pre-treatment sessions (Weeks 9–11) was compared to the last 3 pre-treatment sessions (Weeks 18–20) to estimate the magnitude of untreated recovery; and performance on the last 3 pre-treatment sessions were compared to the final treatment sessions (Weeks 51–54) to estimate treated recovery. Effect sizes were calculated to provide a standardized index of change during the untreated and treated time periods using the $d$ statistic calculated according to procedures described by Busk and Serlin [10] (equation 1).[1] For reading rate, this yielded an effect size of 2.69 comparing the beginning and end of the untreated phase, and an effect size of 9.21 comparing the end of untreated phase with the end of the treatment phase (see Table 3). For reading accuracy, the effect size was 0.82 comparing the untreated phases and 2.18 comparing the treated phase to the previous untreated phase. Thus, the magnitude of change in level of performance during the treated phase was about three times that obtained during the untreated phase for reading rate and accuracy. There

[1] The $d$ statistic is calculated as follows:

$$d = \frac{\bar{X}_{A_2} - \bar{X}_{A_1}}{S_{A_1}} \quad (1)$$

Where $A_2$ refers to the second phase (e.g., post-treatment) and $A_1$ refers to the first phase (e.g., pre-treatment), and S refers to the standard deviation from the first phase (pre-treatment). This is Busk and Serlin's [10] first estimator for $d$.

Table 3
Comparison of text reading rate (words per minute) and accuracy (errors per 100 words) early in the untreated phase (Weeks 9–11), at the end of the untreated phase (Weeks 18-20), and following treatment (Weeks 51-54). The d statistic provides a standardized index of the level of change during the untreated and treated phases

| Testing time | # observations | mean | sd | Index of change (d statistic) | Magnitude of effect size* |
|---|---|---|---|---|---|
| Reading rate (wpm) | | | | | |
| Initial assessment Weeks 9–11 | 3 | 21.55 | 5.87 | —- | —- |
| Following No-Treatment Weeks 18–20 | 3 | 37.34 | 2.09 | 2.69 untreated | small |
| Following Treatment Weeks 51–54 | 3 | 56.60 | 9.28 | 9.21 treated | large |
| Reading accuracy (errors/100 words) | | | | | |
| Initial assessment Weeks 9–11 | 3 | 3.00 | 0.77 | —- | —- |
| Following No-Treatment Weeks 18–20 | 3 | 4.33 | 1.53 | 0.82 untreated | minimal |
| Following Treatment Weeks 51–54 | 3 | 1.00 | 0.00 | 2.18 treated | small |

*Magnitude of effect size determined relative to other single-subject treatment studies (see text for details).

is not a standard metric for the interpretation of single case treatment effect sizes, but the magnitude of the obtained effect sizes can be considered relative to benchmarks derived from the aphasia treatment literature. Robey and colleagues [40] reviewed the published single-subject studies of aphasia treatment and calculated the retrievable effect sizes from 12 studies. With one extreme outlier removed, the first, second, and third quartiles for the d statistic were 2.6, 3.9, and 5.8, corresponding to small, medium, and large sized effects, respectively. Relative to those benchmarks, RB's change in reading rate was small during the untreated phase, and large following treatment. Changes in reading accuracy were small during both phases (see Table 3).

Portions of the GORT-3 were re-administered at 39, 46, and 54 weeks post stroke (see Fig. 3). The obtained reading rates were consistent with those obtained for the novel SRA texts, and served to confirm RB's relatively good reading comprehension. At 54 weeks, RB's reading rate was 67.7 wpm on three passages with 1.3 errors and 0.9 self-corrections on the GORT-3 (Form B). RB's reading comprehension was good with an average of 4.7/5 (93%) questions answered correctly per passage.

### 2.9. Single-word reading

Single-word reading was reassessed on several occasions during the treatment phase, but not with adequate frequency to discern the relative rate of change during pre-treatment versus treatment phases. RB's single-word reading accuracy improved to near normal levels by the end of treatment (Table 2) with no residual lexical effects of imageability, frequency, or grammatical class. His single-word reading reaction times improved (i.e., reduced) over the course of treatment to an average of 3 seconds per word. Significant effects of word length on reading reaction time were repeatedly documented until the end of treatment (54 weeks), when it was no longer significant.

### 2.10. Written spelling

Written spelling was reassessed as shown in Table 2, but was not treated. Whereas the pre-treatment testing variously revealed significant effects of imageability, frequency, grammatical class, and regularity, only a regularity effect persisted. Phonologically plausible errors were most prominent, ranging from 64% to 85% of his total errors. At 39 weeks post stroke, additional examination of spelling using a word list controlled for regularity confirmed a marked regularity effect at that time; RB correctly spelled 17/19 (89%) regular words and 7/19 (37%) exception words ($\chi^2 = 11.31$; $p < 0.001$). Overall, RB showed modest improvements in written spelling during the pre-treatment and treatment phases, so that his performance at the end of the year was significantly improved compared to initial performance on written naming on the PALPA 53 ($\chi^2 = 6.65$, $p = 0.01$); however, a profile of lexical agraphia persisted.

### 2.11. Confrontation naming

Confrontation naming was reassessed on several occasions using the BNT. At 29 weeks, RB correctly named 47/60 (78%) of the pictures. His naming ability remained relatively stable over the course of the year, with a score of 47/60 (78%) at 40 and 52 weeks post stroke, demonstrating a mild persistent anomia.

## 3. Discussion

RB experienced a left hemisphere stroke resulting in letter-by-letter reading, lexical agraphia, and mild anomia. His performance was documented over the ensuing year as natural recovery took place up to 20 weeks post-stroke, and reading treatment was implemented from 22 to 54 weeks. RB's letter naming deficit resolved relatively quickly so that it approximated normal performance by 15 weeks post-stroke, prior to the initiation of treatment. Single-word reading accuracy also improved relatively rapidly, reaching 90% during the first 12 weeks post-stroke, and approximating normal levels by the end of treatment. RB's slow and error-prone reading of text was his primary concern and the focus of the treatment. Although RB showed improvement in oral reading accuracy and rate for new text during the period of spontaneous recovery, he showed even greater improvement during the subsequent administration of MOR treatment.

RB's spontaneous recovery of letter naming allowed him to more accurately decode words using a letter-by-letter reading strategy. This untreated recovery of letter naming was similar to patients reported by Hecean et al. [23] (Case 5) and Newcombe et al. [36] (Patient MB) who showed complete recovery of letter naming ability by 6 and 25 weeks, respectively. Hecean's patient also showed a return to relatively accurate, but slow single-word reading, whereas single-word reading accuracy remained impaired in Newcombe's patient even after 120 weeks post-stroke. Other LBL readers have shown persistent impairment of letter naming, so that tactile-kinesthetic treatment approaches were employed to improve letter recognition and thus improve the accuracy of LBL reading [28,29]. In the case of RB, letter naming and single word reading accuracy similarly showed spontaneous recovery, but the residual visual errors and slow reading rate for text remained a persistent problem. Of course, explicit letter naming is not necessary for whole word recognition under normal circumstances, or even in the case of alexia due to central language impairment wherein individuals are able to read words although they cannot name the component letters. In contrast, letter naming is critical for LBL reading, and despite the fact that it is slow and laborious, it is the first available alternative strategy for those with "pure" alexia.

The rationale for implementing MOR treatment was to improve reading rate by facilitating whole word recognition. The first question of interest was whether there was a therapeutic effect of MOR. We found that RB's improvement during treatment was of greater magnitude than that observed during the extended period of observation prior to treatment. This pattern is not typical of spontaneous recovery alone, which has its steepest slope closer to the onset of impairment. Robey and colleagues [39] provided an index of spontaneous recovery rates in a meta-analysis of within-group studies that measured untreated recovery of aphasia during the acute phase (1 to 3 months), post-acute phase (4 to 12 months post onset), and chronic phase (greater than one year post onset). The average effect size (measured with the d statistic) for change during the acute phase of spontaneous recovery was 0.63 compared to 0.34 during the post-acute phase. Robey's findings suggest that, on the average, the level of change associated with natural recovery during the post-acute phase was half that observed during the acute phase. In comparison, the effect size for RB's change in level of performance following treatment during the post-acute phase was three times the effect size measured during the untreated acute phase. These findings suggest the improvements documented in response to MOR were sufficiently greater than that expected on the basis of spontaneous recovery alone.

The underlying mechanism whereby MOR influences reading is of interest, but is not entirely clear. Moyer [33] proposed that MOR treatment facilitated both bottom-up and top-down processing of text due to practicing word identification in a syntactic/semantic context. It appeared that RB was successful in shifting from serial to whole word processing for text reading, indicating that direct access to the orthographic lexicon improved. Thus, MOR appeared to facilitate visual word form recognition in RB in that reading rate for text and single words improved. Text reading was ultimately easier for RB than reading isolated words, as indicated by the fact that words were recognized in text at a rate of about 1 per second (i.e., 60 words per minute), whereas single word reading still required about 3 seconds per word. Along that line, RB commented after several months of MOR treatment, "I think hearing myself speak helps me maintain a pace."

Repeated measurement of reading reaction times for single words showed a word length effect over the course of the year, suggesting that a serial processing approach was necessary when reading words out of context. Previous research with LBL readers typically has shown improved reading rates with a persistence of the word length effect [2,5,8], suggesting that participants achieved a more rapid implementation of serial processing of letter strings. The final measurements of reading reaction time suggested that the word length effect was negligible for RB by week 54. This was similar to patients SV and TD reported by Beeson & Insalaco [6] who showed resolution of the word length effect when measured at the end of treatment, but data were unavailable to discern when the word length effect resolved. It will be of interest in future studies to test single word reaction times more consistently during MOR treatment to discern whether resolution of the word length effect provides additional information regarding the mechanism for improvement.

It is important to note that RB showed a reduction in reading errors over time, so that reading rate improved without sacrificing accuracy. Thus, despite the fact that MOR explicitly focused on increasing reading rate, it did not result in a speed-accuracy trade-off. From a practical perspective, RB's return to pleasure reading about midway through MOR treatment was confirmation that the treatment effected a change that had a meaningful impact on his life.

Finally, we acknowledge that, although RB showed clinically and functionally significant changes over the course of one year, his reading rate remained slow and effortful relative to normal performance, so that continued treatment was warranted. It is not clear whether RB would gain additional benefit from MOR, had treatment continued. Another possibility is that his residual reading difficulty reflected impairment to the integrity of orthographic representations themselves. In other words, it may be the case that MOR treatment served to improve RB's access to orthographic representations, but reading remained somewhat laborious because those representations were degraded. Given the fact that RB's written spelling was impaired as evidenced by the pattern of lexical agraphia, it is plausible that his residual impairment of reading and spelling reflected damage to a unitary orthographic system. The issue of whether there is a single orthographic lexicon that serves both reading and spelling, rather than separate orthographic input and output lexica, is an issue that has been debated at length elsewhere (e.g., 14,44), and remains unresolved. However, in this context, damage to a single orthographic lexicon provides a more parsimonious explanation of RB's profile than positing concomitant damage to separate orthographic input and output lexica. For spelling, RB relied on preserved phonological processes to compensate for weak orthographic knowledge, as evidenced by the prevalence of phonologically plausible misspellings. RB did not shift to a phonological strategy for reading, most likely because the lexical approach was often successful given additional processing time. It follows that a subsequent treatment for RB might include a protocol directed toward strengthening orthographic representations, that is, a lexical spelling treatment with an expected benefit to both reading and spelling [7].

In summary, our findings with RB add to the data supporting the therapeutic value of MOR to improve reading rate and accuracy for text in individuals with acquired alexia characterized by LBL reading. Research to date suggests that the optimal candidate for MOR may be an individual with intact or recovered letter processing ability who exhibits word length effects and relatively intact orthographic knowledge. For such individuals, it appears that MOR serves to facilitate a shift from serial LBL processing to reading by whole-word recognition. However, given that LBL readers are not a homogeneous group, future studies should aim to further characterize the influence of individual variables on response to MOR treatment.

## Acknowledgement

This research was supported in part by the National Multipurpose Research and Training Grant DC-01409 from the National Institute on Deafness and Other Communication Disorders and the National Science Foundation Graduate Research Training Grant DGE-9355028 to the second author.

The authors thank RB for his participation in this research, and Dr. Steven Rapcsak for referring him to our program.
